# Screening of Early Flowering Lotus (*Nelumbo nucifera* Gaertn.) Cultivars and Effects of Different Cultivars on Flowering Period

**DOI:** 10.3390/plants12081683

**Published:** 2023-04-17

**Authors:** Huiyan Jiang, Junjie Chen, Guangyang Liu, Ping Zhou, Qijiang Jin, Yanjie Wang, Huan Guo, Ping Qian, Yingchun Xu

**Affiliations:** 1 Key Laboratory of Landscape Agriculture, Ministry of Agriculture and Rural Affairs, Key Laboratory of Flower Biology and Germplasm Development, Ministry of Agriculture and Rural Affairs, Key Laboratory of Biology of Ornamental Plants in East China, National Forestry and Grassland Administration, College of Horticulture, Nanjing Agricultural University, Nanjing 210095, China; 2 Zhejiang Weida Garden Engineering Company, Hangzhou 311201, China; 3 Hangzhou West Lake Scenic Area Management Committee, Hangzhou 310013, China

**Keywords:** *Nelumbo nucifera*, cultivar screening, temperature, flowering time, phenological period

## Abstract

Flowering time is an important trait that determines the breeding process of ornamental plants. The flowering period of lotus (*Nelumbo nucifera* Gaertn.) is mainly concentrated in June–August. During this period, the weather is hot and there are few tourists, which made many lotus scenic spots difficult to operate. People have a strong demand for early flowering lotus cultivars. In this paper, 30 lotus cultivars with high ornamental value were selected as materials and their phenological periods were observed for two consecutive years in 2019 and 2020. A number of cultivars with early flowering potential and stable flowering periods, such as ‘Fenyanzi’, ‘Chengshanqiuyue’, ‘Xianghumingyue’ and ‘Wuzhilian’, were screened by K-Means clustering method. The relationship between accumulated temperature and flowering time of 19 lotus cultivars at different growth stages was analyzed. It was found that lotus cultivars with early flowering traits could adapt well to the changes of early environmental temperature and were not affected by low temperature. On the other hand, by analyzing the relationship between different traits and flowering time of three typical cultivars, such as rhizome weight, phenological period, etc., it shows that the nutrient content of the rhizome and the early morphology of plants will affect the flowering time. These results provide a reference for the formation of a systematic lotus early flowering cultivar breeding mechanism and the establishment of a perfect flowering regulation technology system, which can further improve the ornamental value of lotus and promote industrial development.

## 1. Introduction

Lotus (*Nelumbo nucifera* Gaertn.) is a perennial aquatic herb of the genus Nelumbo in the family Nelumbonaceae, and is one of the traditional Chinese famous flowers with high ornamental, edible and medicinal value [1,2]. It is a special crop in which vegetative growth and reproductive growth are alternated and nutrients are provided by propagules in the early growth stage [3]. The flowering period of common lotus cultivars is concentrated in summer from June to September [4], which limits people’s enthusiasm for appreciating lotus, and people have a strong demand for lotus cultivars whose flowering period is extended, from spring to autumn. At present, the method of greenhouse heating and early planting is used to realize the early flowering of lotus. This method consumes a lot of energy and has high production cost. It is urgent to cultivate lotus cultivars with early flowering and stable flowering period from the perspective of cultivar improvement.

It is generally believed that ambient temperature has an important effect on flowering time [5]. Most plants bloom early in the face of a warm environment [6]. In Arabidopsis, elevated temperature from 23 °C to 27 °C can up-regulate FT (FLOWERING LOCUS T) expression and promote flowering even under non-induced SD (Short day) conditions [7,8]. The appropriate temperature is very important for the growth and development of lotus. The germination of terminal buds, the growth of underground stems, the formation of flower buds and the synthesis of organic nutrients are all regulated by temperature [9]. Plant flowering needs to complete certain accumulated temperature requirements. Accumulated temperature has become an important indicator to measure the demand for temperature conditions for plant growth and development. With climate warming, the accumulated temperature requirements for flowering of most woody plants in the northern hemisphere are met faster in spring, resulting in a significant advance in the beginning of flowering [10,11]. Chen [12] found that elevated soil temperature would lead to an increase in soil effective accumulated temperature, and the effective accumulated temperature was linearly related to the germination progress of *Fraxinus chinensis*. Major genetic loci related to flowering under accumulated temperature response were identified in soybean populations [13]. There are few reports studying the flowering period of lotus through accumulated temperature, but systematic studies are still lacking [14].

Sucrose can promote early flowering of some plants [15]. As the reservoir organ in the source-pool theory, flower organs need constant nutrient supply from source organs during their development [16,17,18], the source-sink relationship was closely related to plant growth and development [19]. Pseudobulbs of Orchidaceae plants are important storage organs for water, carbohydrates and minerals. Therefore, pseudobulbs play a key role in nutrient supply, regulation of photosynthesis, flower development and fruit ripening and other vegetative and reproductive growth stages [20]. Lotus rhizome as the source organ can affect the early growth and development behavior of the plants, especially the distribution and utilization of nutrients. Combined with the branching characteristics of the plants themselves, this results in different flowering stages. During the process of rhizome germination, the rhizome used its own nutrients to support plant morphogenesis, mainly promoting the formation of photosynthetic organs, and the appearance of leaves marks the transformation of plants from heterotrophic state to autotrophic state. The difference in rhizome quality and the acceleration of germination speed provides ideas for advancing the initial flowering period of lotus in cultivation techniques.

In the production practice of several consecutive years, there are a number of potted lotus cultivars that can open the first flower early in mid-May under natural conditions, with the characteristics of early flowering. The phenomenon of early flowering of these cultivars may not be retained by asexual reproduction. Whether it belongs to the characteristics of the cultivars themselves or caused by the special meteorological conditions in a certain year remains to be explored. On the basis of identifying its early flowering characteristics, the relationship between its growth and development stages under natural conditions and environmental factors (mainly temperature and light) can be further analyzed to select materials that are easy to control in production. On the one hand, it can greatly reduce the energy consumption of greenhouse heating; on the other hand, the combination of early flowering cultivars and conventional cultivars will prolong the natural flowering period of lotus and increase the supply time of lotus, which is of great significance to promote the development of lotus industry.

In this study, the flowering period of 30 lotus cultivars was observed and classified. Among the cultivars with early flowering ability, the screening analysis was objectively carried out to reveal the relationship between the flowering time of lotus and the accumulated temperature, so as to provide theoretical basis for the screening and application of early flowering cultivars. On this basis, the lotus cultivars with different flowering periods were further observed to explore the reasons for the differences in flowering periods of different cultivars, and to provide a theoretical basis for determining early flowering cultivars.

## 2. Results

### 2.1. Screening Results of Early Flowering Lotus Cultivars in 2019–2020

In order to screen out early flowering lotus cultivars, we conducted a two-year phenological observation of 30 lotus cultivars in Xiaoshan area of Zhejiang Province to obtain the data of average first flowering time, and further classified the cultivars with early flowering potential according to K-Means clustering method. For 2019 the results of the 106 analyses are shown in Table 1: according to the average first flowering time, 30 lotus cultivars were artificially set up in three clusters, which were divided into three categories: early flowering, intermediate flowering and late flowering. 10 cultivars were classified as early flowering, 15 cultivars were classified as intermediate flowering and 5 cultivars were classified as late flowering, indicating that there were indeed differences between different cultivars. Among them, the days from planting to flowering of ‘Baijuhua’ and ‘Fenyanzi’ were significantly less than other cultivars.

**Figure 1:**
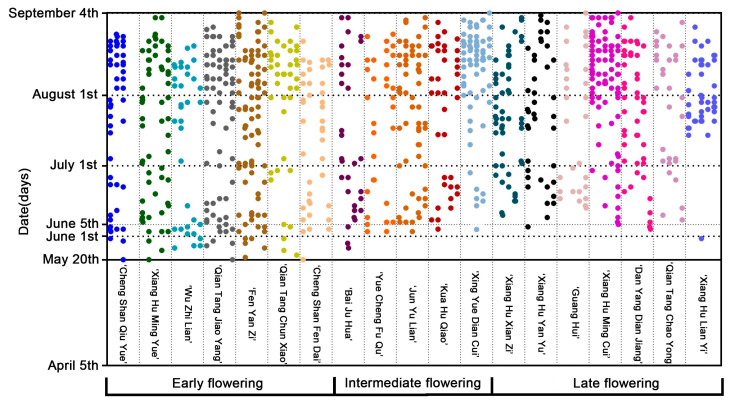
Scatter plots of blooming time of 19 cultivars in 2020.

Due to the selection of early flowering cultivars, in line with the principle of “early selection in the early, excellent selection in the excellent”, in the 2020 screening experiment, most of the intermediate flowering and late flowering cultivars with insufficient provenance, poor ornamental value, and large individual differences were eliminated. In 2020, phenological period observation was carried out on the selected 19 cultivars. The final clustering center in 2019 was used as the initial clustering center in 2020, and 19 cultivars were clustered into 3 categories (Table 2). There were 7 cultivars in the early flowering group, 5 cultivars in the intermediate flowering group, and 7 cultivars in the late flowering group. In addition, by drawing the scatter plot of the flower period, we observed that the blooming period of the 2020 lotus began on May 20, and several early flowering cultivars such as ‘Chengshanqiuyue’, ‘Xianghumingyue’, and ‘Qiantangjiaoyang’ all opened their first flowers on this date (Figure 1). Based on two years of screening, a group of early flowering potential cultivars of ‘Fenyanzi’, ‘Chengshanqiuyue’, ‘Qiantangchunxiao’, ‘Xianghumingyue’ and ‘Wuzhilian’ has been identified, which can be used for subsequent research to promote production.

### 2.2. Weather Differences in 2019–2020 Caused Different Flowering Periods

**Figure 2:**
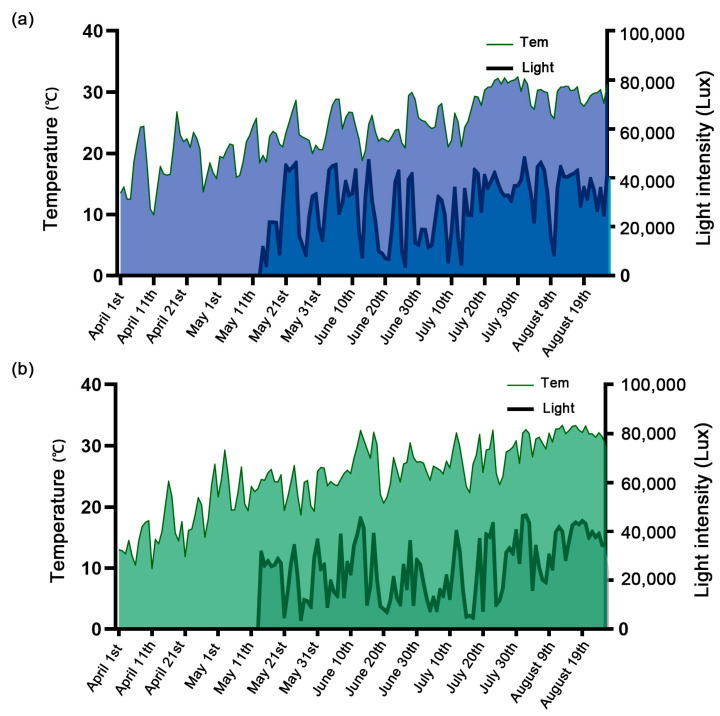
Diurnal variation of average light and temperature in 2019 and 2020. (**a**) Local average temperature and light in 2019. (**b**) Local average temperature and light in 2020.

We observed that the average initial flowering time of some cultivars was different between the two years, sometimes significantly different, indicating that the early flowering characteristics of some cultivars, such as ‘Baijuhua’, ‘Guanghui’, ‘Xianghulianyi’, etc., were affected by environmental factors. However, it is obvious that the flowering period of most cultivars in 2020 is later than that in 2019 (Table 1 and Table 2). Therefore, we obtained daily temperature and illuminance through sensors, and described the overall trend of temperature and illuminance changes in 2019 and 2020 (Figure 2). It showed a gradual upward trend from April to September. The average temperature in April was greater than 10 °C, greater than 15 °C in May, and greater than 20 °C in June. The trend of temperature and illumination in 2020 is roughly the same as that in 2019, and the temperature showed a gradual upward trend. The difference between the two years is that the temperature in April 2020 is only a greater increase on April 15, so that the average temperature in April is lower than that in 2019. According to the statistics of light intensity in the two years, it is found that the light intensity can reach 40,000 lux on days with better light conditions, while it is below 20,000 lux when the light is weak. The difference between the light intensity in the two years is due to the more precipitation from mid-May to mid-June 2020, hence resulting in fewer days with light intensity above 40,000 lux.

### 2.3. Variation of Accumulated Temperature Required by 19 Cultivars during Two-Year Growth Period

**Figure 3:**
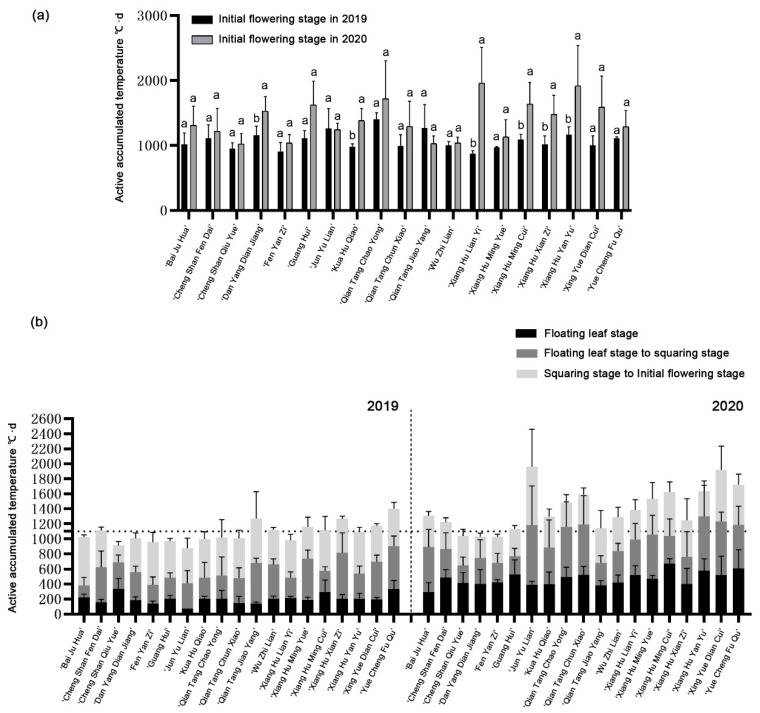
Comparison of accumulated temperature required for each growth period of 19 cultivars in 2019 and 2020. (**a**) Accumulated temperature required for initial flowering stage in 2019 and 2020. (**b**) Accumulated temperature required for each growth period in 2019 and 2020. Different lower case letters mean significant difference at *p* = 0.05.2.4. Flowering Identification of Three Typical Cultivars.

By counting the changes of accumulated temperature during the two-year growth period, it was found that most cultivars could complete flowering below 1100 °C · d in 2019, while they generally needed more than 1100 °C · d to flower in 2020 (Figure 3a). Further analysis found that the main difference in the required accumulated temperature between the two years came from the floating leaf period. The active accumulated temperature required for most cultivars to expand floating leaves doubled in 2020 (Figure 3b). Combined with the meteorological data observed in the field, it may be due to two large increases in temperature from April to May 2019, which promotes the nutrient decomposition of rhizomes and enables plants to complete the expansion of floating leaves with a lower accumulated temperature. It shows that the growth and development rate of plants before the floating leaf stage is greatly affected by temperature. Whether in 2019 or 2020, the accumulated temperature required from the floating leaf stage to the erect leaf stage varies greatly among cultivars, indicating that this stage is mainly affected by its own cultivar characteristics. The accumulated temperature required for flowering of ‘Fenyanzi’, ‘Wuzhilian’, ‘Chengshanqiuyue’ and ‘Xianghumingyue’ showed a low and stable trend in two years, which may be the main reason for the early flowering characteristics.

**Figure 4:**
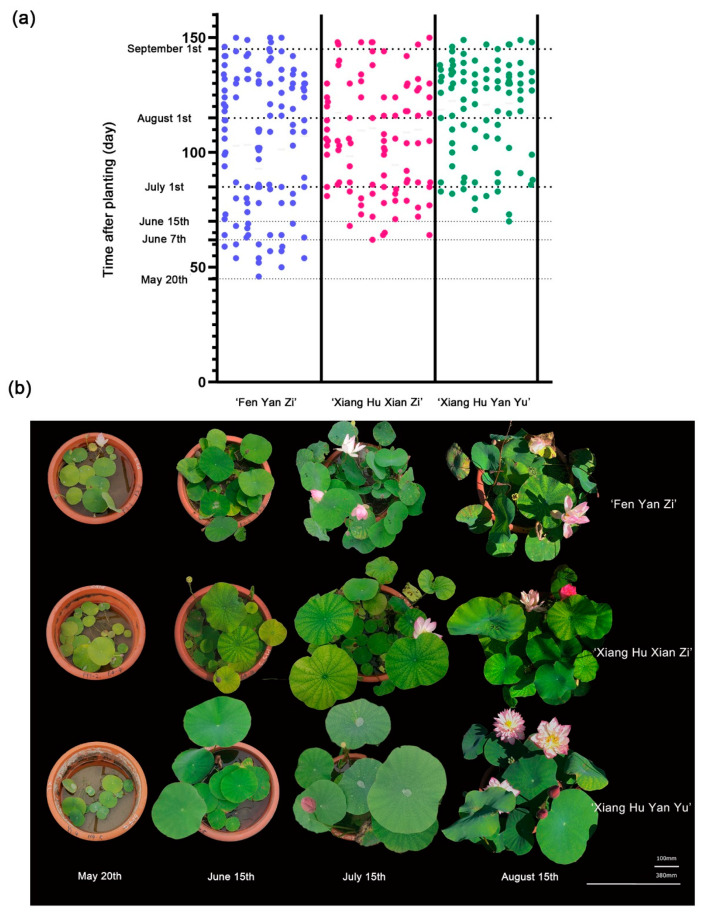
Flowering time of three typical cultivars in 2020. (**a**) Scatter plots of blooming time of three cultivars. (**b**) The growth and development of three cultivars in four periods.

After two years of screening experiments and cluster analysis, 30 cultivars were classified according to the flowering time. Three cultivars of ‘Fenyanzi’, ‘Xianghuxianzi’ and ‘Xianghuyanyu’ with sufficient materials and different flowering periods were selected to study the causes of early flowering time. After expanding planting samples of the three cultivars in 2020, the third time of flowering was counted and physiological data were measured. First, it can be seen from the flowering scatter plot Figure 4a. The initial flowering period of ‘Fenyanzi’ is between May 20 and June 7, that is, 45–62 days after planting, which is significantly earlier than the other two cultivars. During this period, most plants of ‘Fenyanzi’ have opened the first flower. The initial flowering period of ‘Xianghuxianzi’ was earlier than that of ‘Xianghuyanyu’ from 7 June to 15 June, that is, 62–70 days after planting, 50% of the plants of ‘Xianghuxianzi’ opened the first flower. At this time, ‘Xianghuyanyu’ opened the first flower of all plants. After increasing the sample, it can be seen from the scatter diagram that there are obvious differences in the flowering period of the three cultivars, which is consistent with the classification and evaluation results above (Table 3). Secondly, the differences in growth and development and the average initial flowering time were determined by Figure 4b. It was determined that the three cultivars had differences in flowering and belonged to three groups of early, intermediate and late flowering, respectively. The three cultivars can be used to compare the physiological indexes and morphological indexes.

### 2.4. The Changes of Accumulated Temperature Required for the Growth Period of Three Cultivars in Two Years

**Figure 5:**
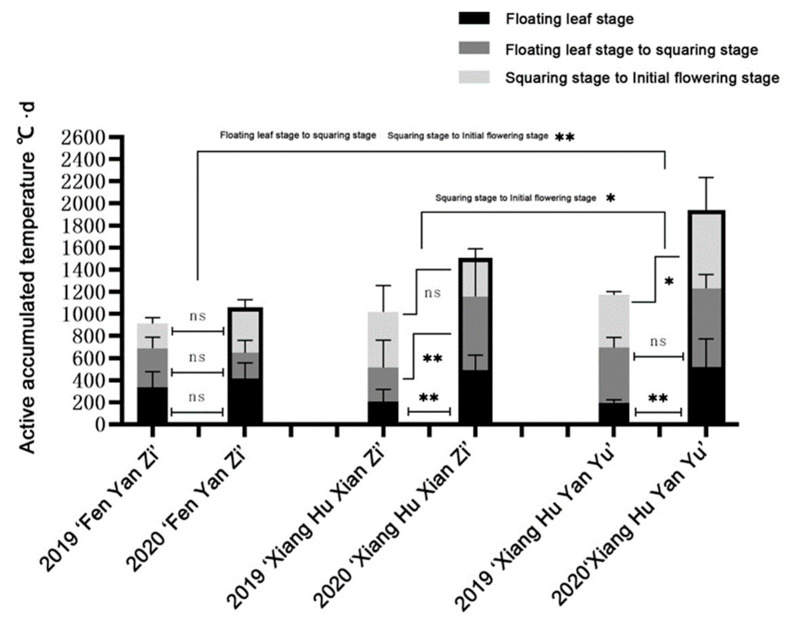
Comparison of accumulated temperature required in each growth period of three cultivars in 2019 and 2020. Asterisks denote *t*-test significance: * *p* < 0.05 and ** *p* < 0.01. ns, not significance.

Three cultivars with different flowering periods were selected as typical cases to further explore the changes of accumulated temperature during the two-year growth period (Figure 5). First of all, there was no significant difference in the accumulated temperature required for the initial flowering period of the three cultivars in 2019. Both ‘Fenyanzi’ and ‘Xianghuxianzi’ can bloom at an accumulated temperature of 1000 °C · d, and ‘Xianghuyanyu’ needs to be close to 1200 °C · d. Subsequently, it was found that the accumulated temperature required for different periods in 2020 was different from that of different cultivars in 2019. There was no significant difference in the accumulated temperature required for the three periods of ‘Fenyanzi’, which better maintained its early flowering characteristics. However, ‘Xianghuxianzi’, which was evaluated as an early flowering cultivar in 2019 and an intermediate flowering cultivar in 2020, showed a significant increase in the accumulated temperature required for the floating leaf period and the accumulated temperature required for the floating leaf period to the squaring period in 2020, which was almost double that of 2019. The late flowering cultivar ‘XianghuYanyu’ also showed a very significant difference that the accumulated temperature required for the floating leaf period in 2020 was more than twice that in 2019. There were obvious differences between intermediate flowering cultivars and late flowering cultivars from squaring stage to initial flowering stage. The late flowering cultivars and early flowering cultivars showed very significant differences in the floating leaf stage to the squaring stage and the squaring stage to the initial flowering stage. This indicated that the sensitivity of different cultivars to temperature was different, which could also lead to early or delayed flowering.

### 2.5. Cause Analysis of Early and Late Flowering of Three Typical Cultivars

First of all, the rhizome provides the nutrients needed for plant growth before the leaf expands, and the accumulated nutrients will directly affect the plant germination, floating leaf expansion time and bud formation. In this study, the rhizomes of the three cultivars were statistically analyzed. It was found that compared with the other two cultivars, the rhizomes of ‘Fenyanzi’ had fewer lateral buds, higher swelling degree and larger fresh weight (Table 4).

Subsequently, by recording the specific time of several stages such as germination stage, floating leaf stage, erect leaf stage and squaring stage, we can conclude that the budding time of ‘Fenyanzi’ is earlier than that of ‘Xianghuxianzi’ and’Xianghuyanyu’ (Table 4), indicating that the difference in flowering time among the three cultivars is due to the difference in squaring stage. In addition, we were surprised to find that bolting time and flowering time of ‘Fenyanzi’ was significantly earlier than the erect leaf expansion time, while the bolting time and flowering time of ‘Xianghuxianzi’ was earlier than the erect leaf expansion time. There was no significant difference between the leaf expansion time and the bud time of ‘Xianghuyanyu’ (Table 5).

Finally, we studied the flower and leaf morphological indexes of the three cultivars (Table 6). It can be seen that ‘Fenyanzi’ is a type of short erect leaf, medium leaf area and short plant type, ‘Xianghuxianzi’ is a type of high erect leaf, small leaf area and thin plant type, ‘Xianghuyanyu’ is a cultivar of high erect leaf, large leaf area and large plant type. These results indicated that the difference in rhizome quality in the early stage and nutrient distribution in the middle stage led to the difference in morphogenesis, which eventually led to the difference in flowering time between cultivars.

## 3. Discussion

As a perennial aquatic flower, the proportion of early flowering cultivars is small, which seriously limits the ornamental period of lotus [21]. Breeding early flowering cultivars is conducive to reducing the cost of flowering regulation and annual production, which is of great significance to production. Wang [22] identified the lotus that bloomed before June in Wuhan as early flowering, those that bloomed before 15 June as intermediate flowering, and those that bloomed after June as late flowering. However, at present, the screening of early-flowering cultivars mostly focuses on the observation of phenological periods [23], and it is impossible to determine whether the early-flowering trait of this cultivar is caused by the cultivar itself or by accidental events induced by environmental factors. Therefore, the early flowering cultivars determined based only on the results of phenological period observation are still unstable in actual production and application. Therefore, in this study, a two-year phenological observation of lotus cultivars was carried out to calculate the effective accumulated temperature and breed cultivars that always maintain early flowering characteristics in different environments. A number of cultivars with early flowering potential and stable flowering period, such as ‘Fenyanzi’, ‘ChengshanQiuyue’, ‘XianghuMingyue’ and ‘Wuzhilian’, have been screened (Table 1 and Table 2, Figure 1), which can be used for subsequent experimental design and production applications.

Previous studies have shown that the transition from vegetative development to reproductive development is controlled by temperature, photoperiod, hormone status and available nutrients [24]. Lotus is a seasonal flower, and its growth and development are mainly affected by temperature and light [25]. In this study, we used sensors to monitor meteorological data for two years (Figure 2), combined with the effective accumulated temperature of 19 lotus cultivars for two consecutive years. Further analysis showed that under the condition that the temperature in 2020 was significantly lower than that in 2019, the four cultivars of ‘Fenyanzi’, ‘Wuzhilian’, ‘Chengshanqiuyue’ and ‘Xianghumingyue’ steadily showed early flowering characteristics in both years (Table 1 and Table 2, Figure 3), In this study, we used sensors to monitor meteorological data for two years (Figure 2), combined with the effective accumulated temperature of 19 lotus cultivars for two consecutive years. The four cultivars were divided into two situations: one is that the accumulated temperature required for each period of ‘Fenyanzi’ does not change between years, indicating that the flowering period of this cultivar is relatively insensitive to temperature changes between different years; the other is that the three cultivars of ‘Wuzhilian’, ‘Chengshanqiuyue’, and ‘Xianghumingyue’ showed an increase in the accumulated temperature required for the floating leaf period, but the accumulated temperature required from the squaring period to the initial flowering period was reduced, such that the accumulated temperature required to finally reach the initial flowering period remained unchanged, maintaining the stability of their own flowering period. Indicating that the three cultivars at the initial stage of germination, like most cultivars, were also affected by the ambient temperature, but by adjusting their own material nutrition distribution, flower bud differentiation began at a lower accumulated temperature so as to flower as early as possible to maintain a stable flowering period. These two types of early flowering cultivars meet the requirements of early flowering and stable early flowering characteristics, and are excellent cultivars for flowering regulation.

The rhizome can be used as a vegetative organ to provide nutrients for plant flowering and breeding, On the other hand, it can also exist as a reproductive organ, which is essential for the growth and development of lotus [26]. There are obvious differences in the flowering period of different lotus cultivars, so the causes of this difference are worth exploring. In the current study, we observed the changes of three typical cultivars of ‘Fenyanzi’, ‘Xianghuxianzi’ and ‘Xianghuyanyu’ during the growth and development period, and explored how different cultivars acted on the flowering period of lotus (Figure 3, Table 3). Our research data showed that ‘Fenyanzi’ has the characteristics of fewer lateral buds, higher expansion of the first and second rhizomes, and greater weight, indicating that the cultivar may store the accumulated nutrients in the expanded underground stems. After the end of dormancy, sufficient nutrients are rapidly supplied to itself for growth, so it can bloom earlier. The fewer branches can reduce the dispersion of different nutrients, help the terminal buds to elongate rapidly and enter the flowering stage (Table 4).

The growth of lotus, no matter seed sowing or rhizome propagation, no matter on top buds or lateral buds, always grows floating leaves first and then erect leaves at the beginning of growth [27,28]. The emergence of erect leaves often represents the transformation of plants from vegetative growth to the coexistence of vegetative growth and reproductive growth. The two leaves also have morphological differences in anatomy. The petiole of erect leaves is upright, so that it can stand out of the water, the leaf area becomes larger and thicker, and the chloroplast development is better than that of floating leaves [29]. Through cytological and comprehensive omics analysis, it was found that the difference in cellulose, lignin content and cell wall structure caused by the difference in polysaccharide synthesis in the cell wall of the two leaves played an important role in the difference in hardness between the two leaves [30]. Therefore, in general, the formation of erect leaves indicates that the nutrient accumulation is relatively sufficient, and the photosynthetic capacity of the position where the erect leaves are formed is significantly stronger than that of the previous section. Comparing the phenological periods of the three cultivars, it was found that the difference in flowering time may come from the squaring stage (Table 5). By comparing the sequence of erect leaves expansion and flower bud appearance, it is speculated that the difference in flower bud appearance time may be related to the amount of nutrients allocated to the leaf. Through field observation, it was found that the erect leaves and flower buds on the same section occurred at the same time and there was no sequence. Both of them were sink organs, and there may be a competitive relationship between them. The time of erect leaves in the early bud cultivar ‘Fenyanzi’ was significantly delayed (Table 5). By comparing the sequence of leaf expansion and bud appearance, it is speculated that the difference in bud appearance time may be related to the amount of nutrients allocated to the leaf. Combined with the indexes of rhizome quality and phenophase (Table 4, Table 5 and Table 6), this phenomenon of delayed flowering and reduction may be related to the nutrient level in the plant. The rhizome of ‘Fenyanzi’ accumulated more nutrients, the plant was short, and the nutrient supply was more concentrated, so that the flower buds of the underground stems quickly flowered in the early stage, forming an excellent trait of early flowering stability. This phenomenon was also found in orchids, and the source-sink conversion relationship in different periods was significantly related to plant growth and reproduction [31].

## 4. Materials and Methods

### 4.1. Plant Materials and Growth Conditions

From 2019 to 2020, the experiment was carried out in Yiqiao Nursery Experimental Base (N 30°03′43.12″ E 120°12′36.93″) of Weida Garden Engineering Co., Ltd., Xiaoshan District, Hangzhou City, Zhejiang Province, China. The photos of 19 cultivars are shown in Table S1.

### 4.2. Measurement of Rhizomes

Internode length: the length between the two nodes closest to the first and second nodes of the terminal bud. Internode diameter: the maximum diameter of the first and second segments closest to the terminal bud. The rhizome expansion coefficient = rhizome single internode diameter/rhizome single internode length. Rhizome fresh weight: The fresh weight of two to three roots. The length of terminal bud: the length of bud scale from node to terminal bud on rhizome. Apical bud diameter: the widest diameter on the apical bud of rhizome. Measurements were done in 3 replicates for each cultivar.

### 4.3. Observation of Phenological Period

The first floating leaf stretched out of the water as a sign of plant germination. The complete expansion of the first floating leaf is taken as a sign of the beginning of the floating leaf period. The complete expansion of the first vertical leaf is taken as a sign of the beginning of the leaf stage. The first flower bud water as a sign of the beginning of the squaring period. Measurements were done in 3 replicates for each cultivar.

For each cultivar, 3–9 rhizomes with consistent growth were selected and planted into a 535 mm non-porous lotus pot. 5 April was taken as the time of colonization, and the first flower developed and matured, the outer flap loosened, and the channel for insect pollination appeared as a sign of entering the flowering period. The date of the first flower of the same cultivar is called the initial flowering time of the cultivar, and the average initial flowering time of the same cultivar is taken as the average initial flowering time of the cultivar.

### 4.4. Measurement of Leaf Traits and Pedicel Height

A tape measure was used to measure the height of all the vertical leaves, pedicels, and pedicels of each pot plant. The maximum width parallel to the axial vein of the leaf was measured as the leaf width, and the maximum length perpendicular to the axial vein of the leaf was measured as the leaf length. Measurements were done in 3 replicates for each cultivar.

### 4.5. Meteorological Data Monitoring

Sensors (Zhongshengkeji, Dongguan, Guangdong, China) were set up in the field and various planting environments to monitor field data such as local temperature and light. The data were updated once every 1 min and uploaded to the cloud. The data were downloaded and analyzed using SPSS.

## 5. Conclusions

By verifying the flowering period of different lotus cultivars, we screened a series of lotus varieties with early flowering potential, such as ‘Fenyanzi’, ‘Wuzhilian’, ‘Chengshanqiuyue’ and ‘Xianghumingyue’. In addition, it was found that the flowering time of lotus was closely related to the nutrients stored in rhizomes and subsequent nutrient distribution, and provided theoretical basis for breeding early flowering cultivars from morphological and physiological indicators. In the follow-up study, the distribution and transportation direction of nutrients in the rhizomes of lotus and photocontracides generated by leaves can be studied in different periods. We can then explorethe source-sink relationship between different organs or tissues, find specific cultivation methods suitable for different growth periods, and effectively regulate the flowering period of lotus in the future, so as to meet the needs of the market and promote the development of industrialization.

## Figures and Tables

**Table 1:** Average number of days from planting to blooming stage of 30 *Nelumbo nucifera* cultivars during 2019.

Cultivar	Average Number of Days from Planting to Blooming Stage (Day)	Cluster Analysis Results of First Flowering Time (Divided into 3 Categories)	Variation Coefficient of First Flowering Time %
‘Bai Ju Hua’	42.00 ± 10.39 AB	Early flowering	25
‘Fen Yan Zi’	47.50 ± 6.03 AB	Early flowering	13
‘Cheng Shan Qiu Yue’	49.50 ± 3.89 ABC	Early flowering	8
‘Xiang Hu Lian Yi’	49.67 ± 7.74 ABC	Early flowering	16
‘Xiang Hu Ming Yue’	50.17 ± 0.75 A	Early flowering	1
‘Kua Hu Qiao’	50.67 ± 1.75 ABC	Early flowering	3
‘Qian Tang Chun Xiao’	51.25 ± 7.41 ABC	Early flowering	14
‘Wu Zhi Lian’	51.60 ± 2.30 ABC	Early flowering	4
‘Xing Yue Dian Cui’	51.75 ± 6.24 ABC	Early flowering	12
‘Xiang Hu Xian Zi’	52.25 ± 5.56 ABC	Early flowering	11
‘Xiang Hu Fen’	54.83 ± 9.06 ABC	Intermediate flowering	17
‘Xiang Hu Ming Cui’	55.33 ± 3.50 ABC	Intermediate flowering	6
‘Hong Zhuang Pi Yu’	55.33 ± 2.31 ABC	Intermediate flowering	4
‘Karizma’	56.00 ± 9.85 ABC	Intermediate flowering	18
‘Yue Cheng Fu Qu’	56.17 ± 0.75 ABC	Intermediate flowering	1
‘Cheng Shan Fen Dai’	56.20 ± 9.09 ABC	Intermediate flowering	16
‘Guang Hui’	56.33 ± 5.13 ABC	Intermediate flowering	9
‘Qian Tang Jiao Yang’	57.17 ± 5.53 ABC	Intermediate flowering	10
‘Xiang Hu Yan Yu’	57.60 ± 5.03 ABC	Intermediate flowering	9
‘Dan Yang Dian Jiang’	58.33 ± 6.03 ABC	Intermediate flowering	10
‘Xiang Hu Ta Xun’	60.00 ± 3.46 ABC	Intermediate flowering	6
‘Cheng Shan Yu Huan’	61.00 ± 4.40 ABC	Intermediate flowering	7
‘Jun Yu Lian’	62.33 ± 12.74 ABC	Intermediate flowering	20
‘Xiang Hu Liu Xia’	66.67 ± 3.06 B	Intermediate flowering	5
‘Qian Tang Chao Yong’	68.20 ± 4.02 B	Intermediate flowering	6
‘Qian Tang Qiu Yun’	70.67 ± 1.53 B	Late flowering	2
‘Qiu Ri Hong Hua’	74.33 ± 9.81 ABC	Late flowering	13
‘Qiu Xing’	77.33 ± 15.37 ABC	Late flowering	20
‘Pi Zhen Fen’	79.00 ± 5.57 C	Late flowering	7
‘He Tang Xiao Yue’	88.33 ± 19.40 ABC	Late flowering	22

Different capital letters in the same column mean extremely remarkable difference at *p* = 0.01.

**Table 2:** Average number of days from planting to blooming stage of 19 *Nelumbo nucifera* cultivars during 2020.

Cultivar	Average Number of Days from Planting to Blooming Stage (Day)	Cluster Analysis Results of First Flowering Time (Divided into 3 Categories)	Variation Coefficient of First Flowering Time %
‘Qian Tang Chun Xiao’	51.67 ± 6.43 AB	Early flowering	12
‘Cheng Shan Qiu Yue’	52.33 ± 6.66 AB	Early flowering	13
‘Qian Tang Jiao Yang’	52.60 ± 4.98 A	Early flowering	9
‘Xiang Hu Ming Yue’	53.00 ± 7.30 AB	Early flowering	14
‘Fen Yan Zi’	53.20 ± 5.26 AB	Early flowering	10
‘Wu Zhi Lian’	53.20 ± 3.56 A	Early flowering	7
‘Cheng Shan Fen Dai’	53.67 ± 7.51 AB	Early flowering	14
‘Yue Cheng Fu Qu’	58.33 ± 2.31 AB	Intermediate flowering ring	4
‘Bai Ju Hua’	59.33 ± 8.33 AB	Intermediate flowering	14
‘Jun Yu Lian’	62.00 ± 4.00 AB	Intermediate flowering	6
‘Kua Hu Qiao’	67.40 ± 7.77 AB	Intermediate flowering	12
‘Xing Yue Dian Cui’	67.50 ± 8.70 AB	Intermediate flowering	13
‘Guang Hui’	71.67 ± 2.08 AB	Late flowering	3
‘Xiang Hu Xian Zi’	71.67 ± 8.62 AB	Late flowering	12
‘Xiang Hu Yan Yu’	71.67 ± 9.61 AB	Late flowering	13
‘Qian Tang Chao Yong’	71.75 ± 10.21 AB	Late flowering	14
‘Dan Yang Dian Jiang’	74.00 ± 9.49 AB	Late flowering	13
‘Xiang Hu Ming Cui’	79.00 ± 9.64 AB	Late flowering	12
‘Xiang Hu Lian Yi’	101.00 ± 3.46 B	Late flowering	3

Different capital letters in the same column mean extremely remarkable difference at *p* = 0.01.

**Table 3:** Comparison of average number of days from planting to blooming stage of 3 *Nelumbo nucifera* cultivars.

Cultivar	First Flowering Time in 2019	Cluster Analysis Results of First Flowering Time in 2019	First Flowering Time in 2020	Cluster Analysis Results of First Flowering Time in 2020	First Flowering Time in 2020 after Increase the Sample Size
‘Fen Yan Zi’	47.50 ± 6.03 a	Early flowering	53.20 ± 5.26 a	Early flowering	56.5 ± 7.27 a
‘Xiang Hu Xian Zi’	52.25 ± 5.56 a	Early flowering	71.67 ± 9.61 b	Late flowering	72.4 ± 8.13 b
‘Xiang Hu Yan Yu’	57.60 ± 5.03 a	Intermediate flowering	71.67 ± 8.62 b	Late flowering	81.67 ± 6.08 c

Different lower case letters mean significant difference at *p* = 0.05.

**Table 4:** Comparison of rhizomes harvested from three cultivars in the last growth period.

Cultivar	Lateral Bud Number	Fresh Weight of Rhizomes	Expansion Coefficient of the First Nod	Expansion Coefficient of the Second Nod	Length of Apical Bud	Width of Apical Bud
‘Fen Yan Zi’	1.00 ± 0.93 b	34.38 ± 17.11 a	0.74 ± 0.19 a	0.63 ± 0.18 a	2.50 ± 0.77 a	0.45 ± 0.09 b
‘Xiang Hu Xian Zi’	2.00 ± 0.71 a	13.92 ± 8.29 b	0.46 ± 0.10 b	0.39 ± 0.16 b	2.72 ±0.78 a	0.45 ± 0.08 b
‘Xiang Hu Yan Yu’	1.92 ± 0.79 a	19.5 ± 8.69 ab	0.47 ± 0.22 b	0.54 ± 0.16 ab	3.12 ± 0.70 a	0.69 ± 0.10 a

Different lower case letters mean significant difference at *p* = 0.05.

**Table 5:** Comparison of phenological periods of 3 *Nelumbo nucifera* cultivars.

Cultivar	Germination Time	Time of Floating Leaves Unfolding	Time of Vertical Leaves Unfolding	Time of Budding
‘Fen Yan Zi’	12.25 ± 6.67 a	25.38 ± 6.76 a	69.38 ± 23.49 a	37.88 ± 7.2 b
‘Xiang Hu Xian Zi’	12.15 ± 3.34 a	24.08 ± 2.43 a	64.38 ± 10.89 a	55.77 ± 11.09 b
‘Xiang Hu Yan Yu’	15.08 ± 8.61 a	27.67 ± 5.97 a	58.08 ± 22.35 a	61.83 ± 12.3 a

Different lower case letters mean significant difference at *p* = 0.05.

**Table 6:** Comparison of leaf characters and flower stalk height among 3 cultivars.

Cultivar	Average Height of Vertical Leaves	Average Area of Vertical Leaves	Average Height of Flower Stalk
‘Fen Yan Zi’	10.92 ± 4.72 b	110.29 ± 22.71 ab	21.34 ± 2.88 b
‘Xiang Hu Xian Zi’	25.54 ± 8.09 a	92.26 ± 23.56 b	35.36 ± 5.59 b
‘Xiang Hu Yan Yu’	24.36 ± 4.41 a	139.47 ± 45.70 a	42.93 ± 9.35 a

Different small letters means significant difference at 0.05.

## Data Availability

The data that support the findings of this study are available from the corresponding author upon reasonable request.

## Supplementary Materials

The following supporting information can be downloaded at: https://www.mdpi.com/article/10.3390/plants12081683/s1, Table S1: Photos of 19 lotus cultivars.
