# Supplementary material for: Screening of Early Flowering Lotus (Nelumbo nucifera Gaertn.) Cultivars and Effects of Different Cultivars on Flowering Period
Source: Plants (Basel). 2023 Apr 17;12(8):1683. doi: 10.3390/plants12081683 (PMC10140858; doi:10.3390/plants12081683)
Supplement: Supplementary file 1 [file plants-12-01683-s001.zip › plants-2317932-supplementary.pdf]

Table S1. Photos of 19 lotus cultivars

Cultivar	Top view	2-3 days after flowering
‘Qian Tang Chun Xiao’	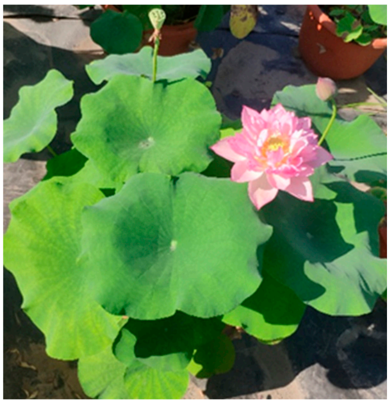	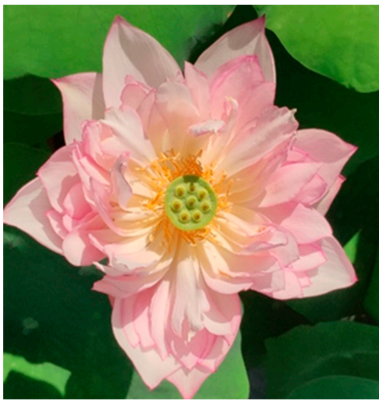
‘Cheng Shan Qiu Yue’	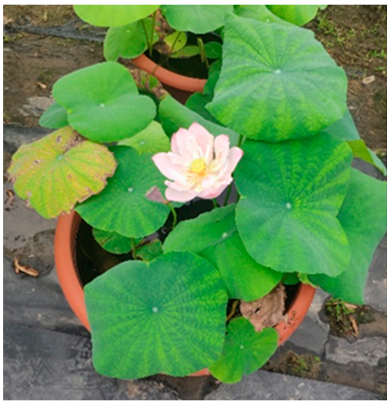	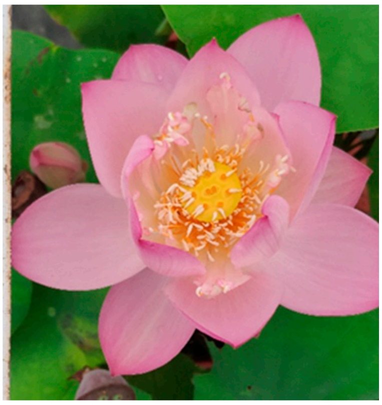
‘Qian Tang Jiao Yang’	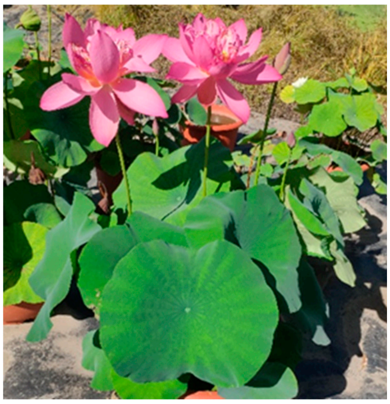	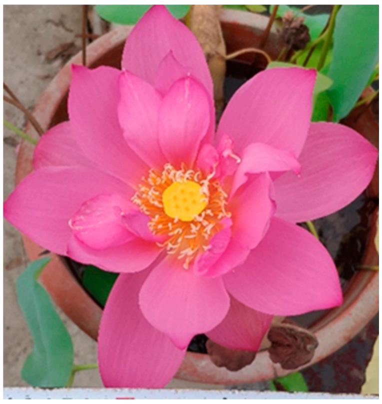
‘Xiang Hu Ming Yue’	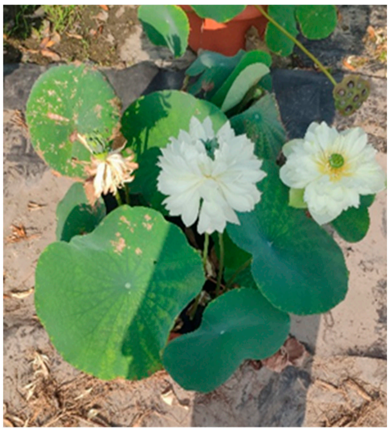	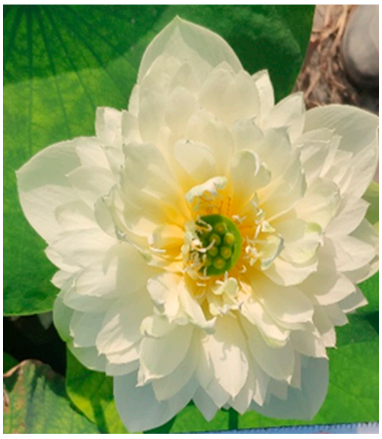

Cultivar	Top view	2-3 days after flowering
‘Fen Yan Zi’	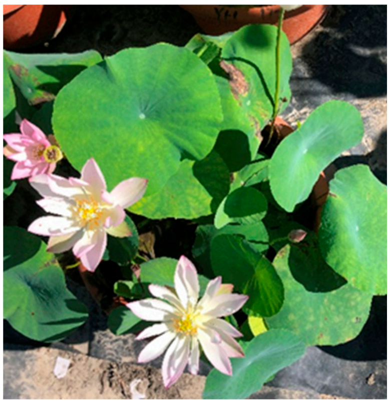	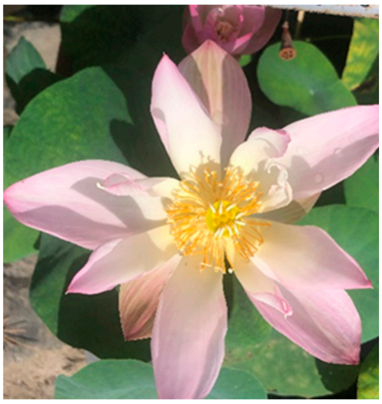
‘Wu Zhi Lian’	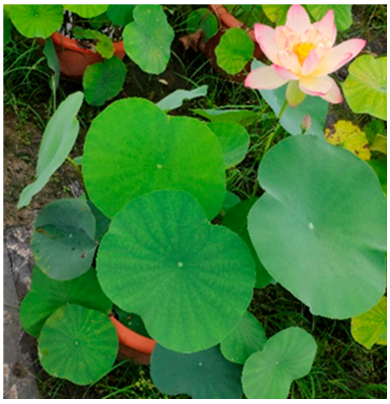	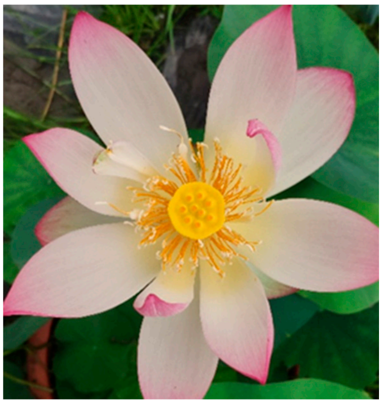
‘Cheng Shan Fen Dai’	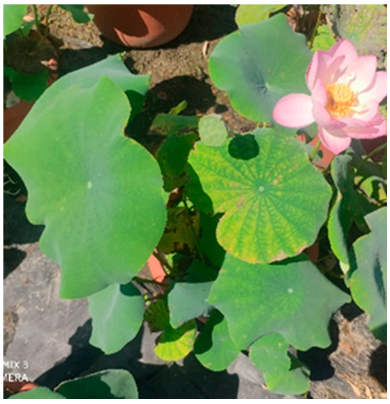	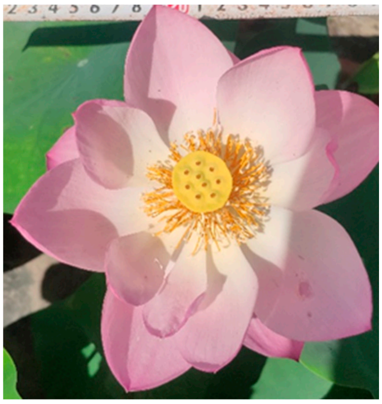
‘Yue Cheng Fu Qu’	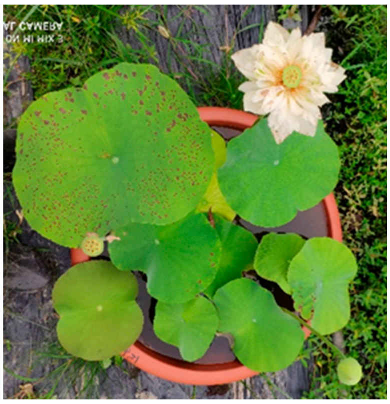	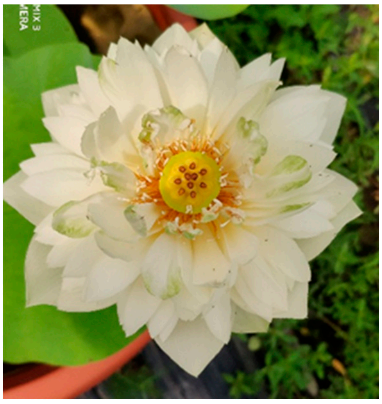

Cultivar	Top view	2-3 days after flowering
‘Bai Ju Hua’	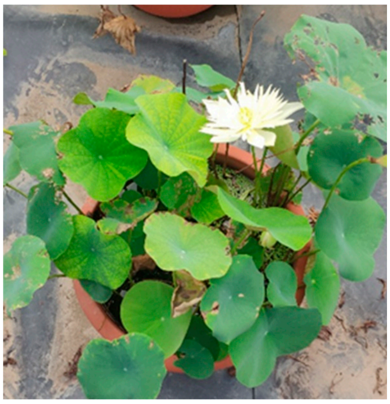	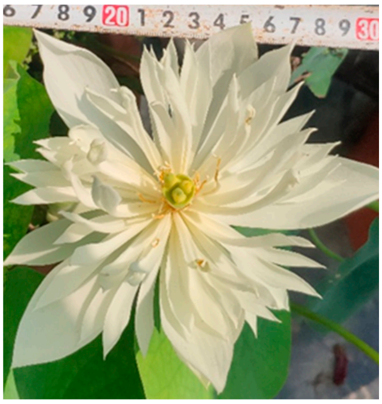
‘Jun Yu Lian’	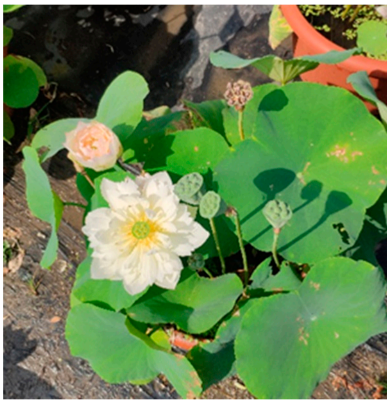	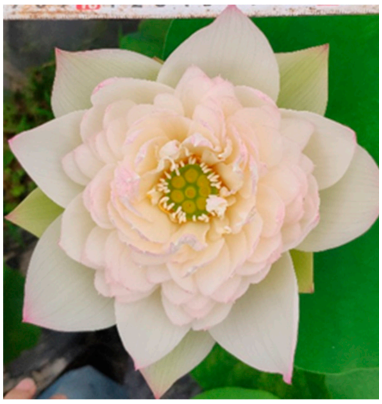
‘Kua Hu Qiao’	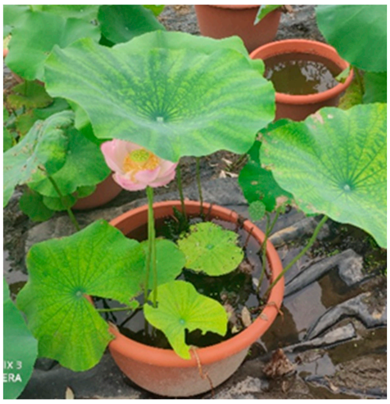	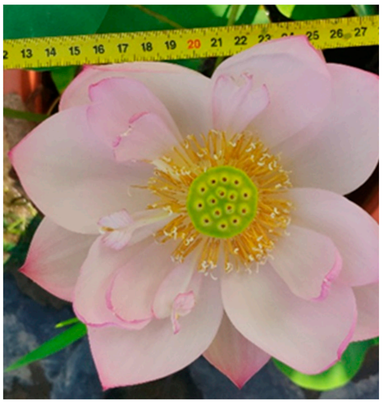
‘Xing Yue Dian Cui’	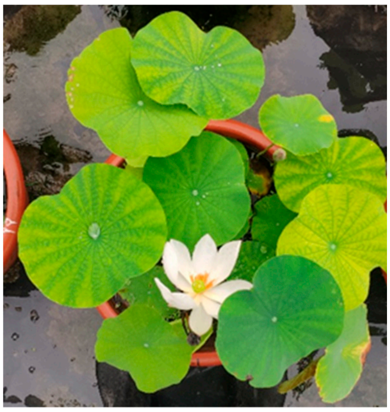	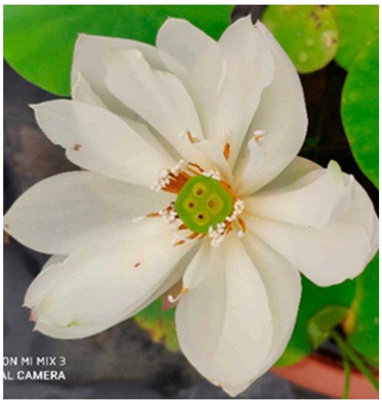

Cultivar	Top view	2-3 days after flowering
‘Guang Hui’	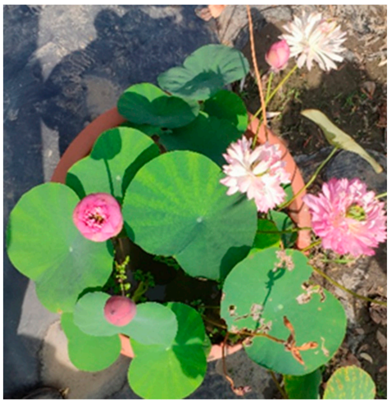	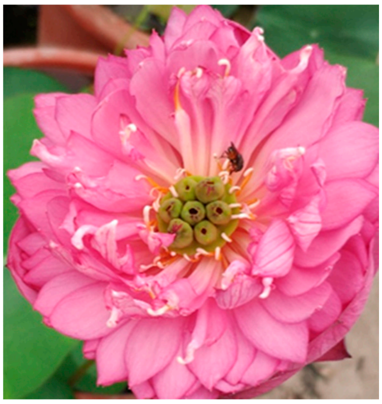
‘Xiang Hu Xian Zi’	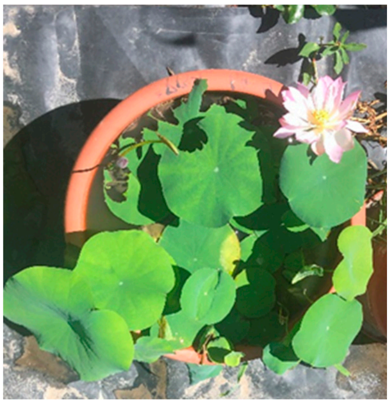	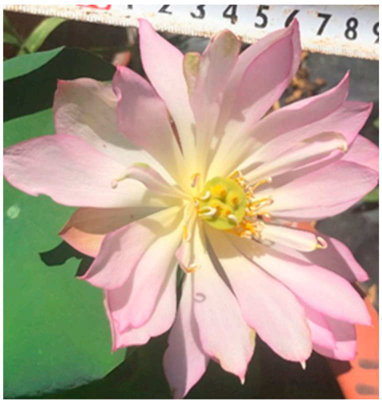
‘Xiang Hu Yan Yu’	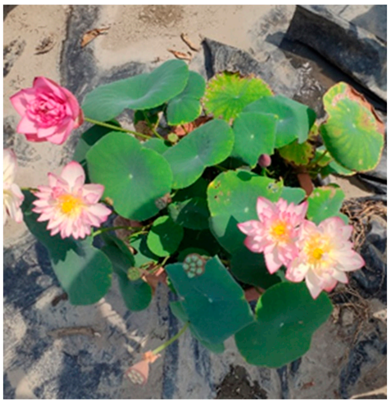	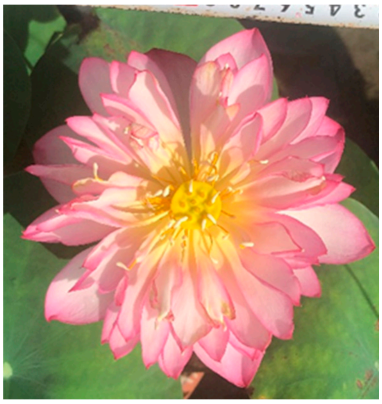
‘Qian Tang Chao Yong’	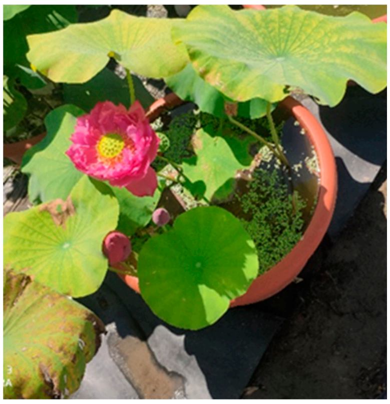	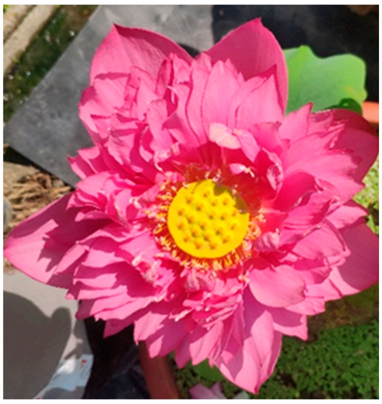

Cultivar	Top view	2-3 days after flowering
‘Dan Yang Dian Jiang’	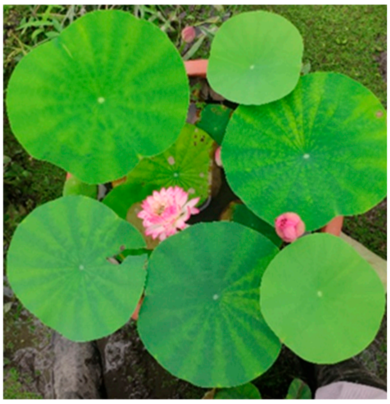	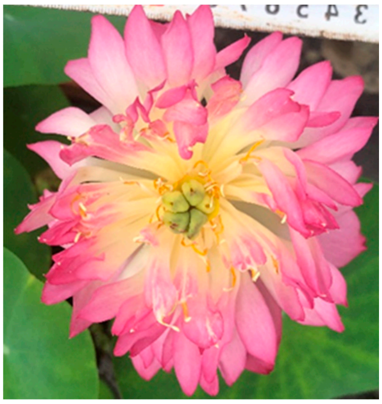
‘Xiang Hu Ming Cui’	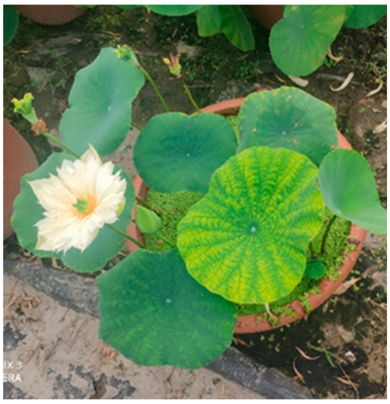	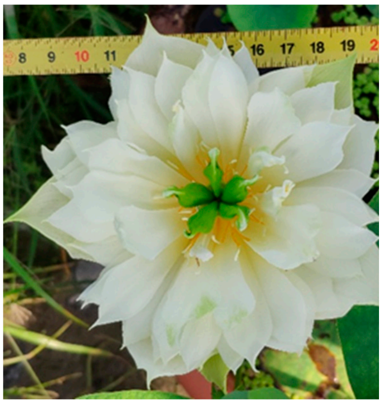
‘Xiang Hu Lian Yi’	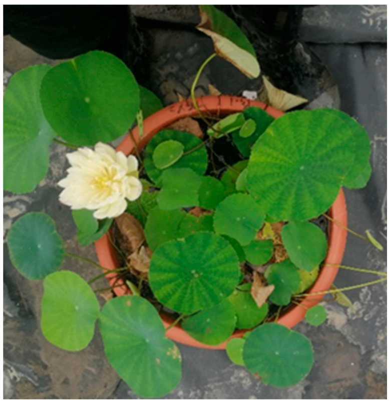	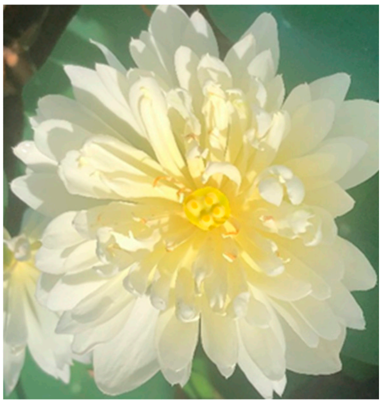
